# Effect of Age, Deboning Time of Carcass, and Different Cooking Conditions on the Woody Breast Myopathies in Chicken: A Meta-Analysis

**DOI:** 10.3390/foods13162632

**Published:** 2024-08-22

**Authors:** Aftab Siddique, Micah T. Black, Bet W. Alvarado, Laura Garner, Tung-Shi Huang, Ashish Gupta, Alan E. Wilson, Jason T. Sawyer, Amit Morey

**Affiliations:** 1 Department of Poultry Science, Auburn University, Auburn, AL 36849, USA; azs0242@auburn.edu (A.S.); mtb0042@auburn.edu (M.T.B.); bsw0033@auburn.edu (B.W.A.); bauerlj@auburn.edu (L.G.); huangtu@auburn.edu (T.-S.H.); 2 Department of Business Analytics and Information, Auburn University, Auburn, AL 36849, USA; azg0074@auburn.edu; 3 School of Fisheries, Aquaculture, & Aquatic Science, Auburn University, Auburn, AL 36849, USA; aew0009@auburn.edu; 4 Department of Animal Science, Auburn University, Auburn, AL 36849, USA; jts0109@auburn.edu

**Keywords:** Blunt Meullenet-Owens Razor Shear, Meullenet-Owens Razor Shear, woody breast fillets, sous vide

## Abstract

This meta-analysis review undertakes a comprehensive examination of various approaches for identifying myopathic fillets and meticulously evaluates the effects of bird age, deboning time, and different cooking and storage conditions on woody breast (WB) myopathic conditions in broiler deboned fillets. The data, meticulously collected from 20 articles based on predefined inclusion criteria sourced from various databases and online resources, reveal significant insights. For instance, the analysis uncovers that deboning time significantly affects Meullenet-Owens Razor Shear (MORS), Blunt Meullenet-Owens Razor Shear (BMORS), and descriptive analysis values (*p* < 0.001). Instrumentation techniques, such as compression force and shear force, along with different cooking conditions, strongly impact BMORS shear force values (R^2^ = 86.80%), with significance levels ranging from 0.01 to 0.001. Deboning time also substantially impacts MORS shear force values (R = 64.03%). In contrast, the effects of deboning time, bird age, and cooking conditions on descriptive sensory evaluation are minimal when compared to woody breast fillets (age of birds: R^2^ = 26.53%; cooking conditions: R^2^ = 32.57%; deboning time: R^2^ = 10.06%). The overall effect of bird age on chicken breast meat quality shows significant differences for the evaluated parameters (Hedges’ g [95% CI] = −0.72 [0.17, 1.26], I^2^ = 93%, *p* < 0.01). The sous vide cooking method significantly affects shear force energies and sensory descriptive evaluation for woody breast fillets (Hedges’ g [95% CI] = 5.30 [−50.30, 83.40], I^2^ = 98%, *p* < 0.01). These findings, with their significant implications, provide valuable insights for optimizing processing conditions in the poultry industry to reduce woody breast occurrences and enhance meat quality, instilling confidence in the robustness of the research.

## 1. Introduction

Different types of meat and meat products that are nutrient-filled are always the first and foremost choice for protein across the world [1]. There has been a drastic increase in meat and meat products consumption worldwide since the last couple of decades. Into which the consumption per capita had increased from 9.99 kg in the 1960s to 25.99 kg in the year 2000 and will reach up to 36.96 kg by the year 2030 [2]. Consumption of meat-based protein is one of the main sources for almost every consumer in the United States as well as at the global level. According to the National Chicken Council (NCC), nearly nine billion broilers were raised in the U.S., and as per estimates, per capita consumption of chicken is nearly 42.86 kg of chicken every year in the United States [2]. The popularity of chicken meat is in high demand because of different organoleptic attributes such as texture, color, and flavors [3]. It can also be acknowledged that broader factors such as nutritional value and affordability also play a significant role in growing demand for poultry meat [4]. High consumer demand for better-quality chicken breast meat is increasing, which is having an impact on the industry to produce fast-growing birds, feed efficiency, and the measurement of the breast muscle [4].

**Figure 1:**
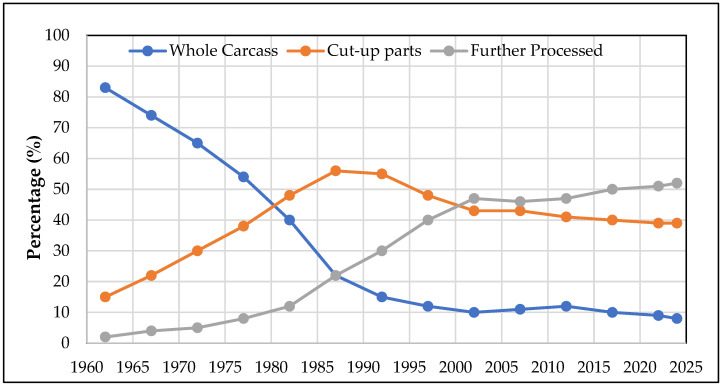
Graphical representation of different percentages of broiler bird market trend from 1962 to 2024 based on whole carcass (blue line), cut-up parts (orange line), and broilers in further processed products (gray line). Data source: National Chicken Council (NCC) [2].

**Figure 2:**
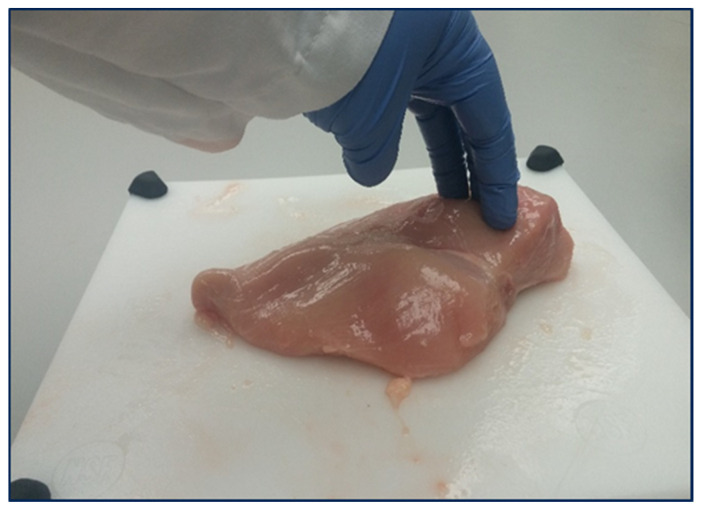
Woody breast representation during sampling.

To meet the excessive demands for boneless white meat, the broiler growers and processors have successfully incorporated and utilized better genetic breed selections, which resulted in improvements in nutritional diet to obtain weight gain in an average chicken, increased growth rate, and also an increase in total carcass yield. In the response of continuously changing market demands, that is completely guided as per end users’ preferences and demands, which are inclined more towards cut-up processed chicken parts than whole chicken carcasses. According to data from the National Chicken Council (NCC) [2], the way broilers are marketed has significantly evolved over the past 60 years. In 1962, 83% of broilers were sold as whole carcasses, with 15% as cut-up parts and only 2% used in further processing. In contrast, today, only 8% of broilers are sold as whole carcasses, while 39% are marketed as cut-up parts, and 52% are now utilized in further processed products. This shift highlights the increasing demand for convenience and value-added poultry products in the market (Figure 1). Despite having fast-growing chickens and an increase in white breast meat yield, there has been an increase in the cases of breast myopathies. One of the muscle abnormalities that has been discovered in broiler breast meat is referred to as “woody breast” and is more dominant in bigger and heavier birds [3]. This woody breast (WB) (Figure 2) condition can be easily identified by their faded-pale color appearance with swollen caudal part of the breast fillet, which consists of varying levels of hard appearance.

The incidence rate of woody breast myopathies in commercial WB myopathy conditions can be identified by stiffness in the breast muscle, which may have on its surface faded pale color and exudate [5,6]. In 2017, a prevalence report published by Sihvo et al. [6] in Finland mentioned the 53% moderate and 12% severe woody breast conditions. Based on the recent report by Chen et al. [7], they have mentioned an 11.8% prevalence rate for woody breast in Ontario, Canada [7]. Velleman et al. [8] stated a theory that the hardness in chicken breast meat may be due to fibrosis, which is the outcome of cross-linked collagen fibril accumulation. Soglia et al. [9] reported that collagen may be one of the reasons for increased firmness related to the development of this condition [9]. These changes in the woody breast muscles can also influence different physical and chemical meat quality attributes such as pH, color, water holding capacity (WHC), cook loss, and texture profile attributes mainly associated with the pectoralis major muscle [10].

### 1.1. Classification Accuracy

In simple terms, classification accuracy can be defined as the number of right predictions divided by the total number of predictions, which highlights the categorization effectiveness of the models. It is the most used statistic for assessing binary classifiers [11]. Classification models have been used in food sciences and related fields for more than a decade. There have been several classification algorithms established and are being used for classification-based studies such as Support Vector Machines (SVM) [12,13,14], Back Propagation Neural Networks (BPNN) [15], and Linear Discriminant Analysis (LDA) [16]. For more details on these classification accuracy models and their implementation, readers can refer to the article Siddique et al. [17].

### 1.2. Compression Force (CF) and Shear Force (SF)

Compression force (CF) can be defined as the force that is being generated by compressing an object or substance. In other words, when shear forces (SFs) aligned with each other are defined as compression forces to the object surface resulting in some degree of deformation. In the poultry processing industry, the textural characteristics of raw chicken breast meat are used as a set of criteria for WB characterization. Several instrumental texture measurements have been used to evaluate the level of WB state in raw chicken fillets, including CF. According to published research, there was a substantial difference in CF between WB and regular normal fillets [17,18]. Mudalal et al. [19] and Soglia et al. [20] found that CF measurements of raw broiler breast meat when compared with the WB state were significantly greater than normal fillets. Since the 1930s, the shear force (SF) test has been the most widely used instrumental approach for measuring meat tenderness [21]. The force (N) as a function of knife movement (mm) and compressed to cut off a sample of tissue is measured in this test (MPa) and is determined by the hardness or toughness of the sample [22]. Shear force denotes the movement of muscle parallel towards the axis of immediate contact while applying tangential force to the section. Nonetheless, within the food industry, this term is widely used to describe any cutting technique that separates a product into smaller fragments [23].

### 1.3. Meullenet-Owens Razor Shear (MORS) and Blunt Meullenet-Owens Razor Shear (BMORS)

Meullenet-Owens Razor Shear (MORS) was developed and first introduced by Cavitt et al. [24], by the name of Razor Blade Shear, which was renamed later as the Meullenet-Owens Razor Shear (MORS) test. They have reported that the use of a razor blade in determining the texture of cooked chicken was much easier and more efficient. In addition to the shear force use of a razor blade on meat samples, it also provides one more additional parameter called “shear energy”. Use of the MORS test on a sample reduces the chances of experimental error, no time is needed to prepare a sample, and MORS is independent of sample size [24]. Meullenet et al. [25] have developed a modified version of MORS that provides better comparison between tough portions of meat [26,27]. Reliability and the effectiveness of BMORS and MORS were demonstrated by Lee et al. [27] on the tenderness of chicken breast meat. Instrumental analysis of breast meat by using the BMORS test had shown much better correlation to the tenderness as reported by the consumer panel [26].

### 1.4. Descriptive Sensory Analysis

Descriptive analysis is one of the methods that elaborates on the quality and intensity of a specific end-user product [25]. A wide range of descriptive analysis techniques have been developed by using the basic principles of sensory science. Conventional descriptive techniques, such as food attribute profiling methods and quantitative descriptive analysis, involve a trained person to objectively quantify the sensory attributes of samples [27]. Due to the versatility of tasks that were completed with descriptive sensory analysis and the amount of generated data, this method had become the valuable source of product information, not just limited to research settings but also for further processed food product development industries and government agencies [26]. Descriptive analysis for quality evaluation of products was first implemented for food products and beverages [25]. The implementation of descriptive sensory evaluation is not only limited to evaluating different attributes of products but is now also being used to monitor product lifecycle, mapping market graphs, variety of product development, value optimization, and quality control of existing line products [25]. Descriptive sensory analysis is more importantly used in various product design and development when sensory data are linked to consumer response through hedonic data and instrumental analysis data for physico-chemical attributes. Relative study of both generated data allows professionals and developers to easily understand the consumer preference trend, which helps companies to design their product and to enhance quality attributes [28].

As the poultry industry is a fast-growing business and can also be considered as one of the important contributing factors in providing food to a growing population, creating jobs, and being consumer-driven in nature, the presented work in this article will provide an insight to the readers about the important parameters to consider for designing their research with set parameters, as different authors have used different parameters of deboning time and storage conditions, along with some insight on the use of big data analytic approaches for the classification of myopathic fillets during inline processing. This paper tries to fill the gap for the literature evidence that can be helpful in designing the experiments and will also be helpful for the poultry processing industries to implement novel techniques for their processes and optimize steps to reduce losses and increase profitability.

## 2. Materials and Methods

### 2.1. Inclusion and Exclusion Criteria

We included studies if they (1) were comparing raw chicken normal fillets with myopathic conditioned fillets; (2) used approaches such as MORS, BMORS, compression force, and shear force; (3) used big data analytics approaches such as Support Vector Machine (SVM), Multilayer Perceptron (MLP), Back Propagation Neural Network (BPNN), and Linear Discriminant Analysis (LDA); (4) used different cooking and storage conditions; (5) had used different processed products made from normal and myopathic fillets; (6) published in English. Additionally, we excluded studies with no comparison between normal and myopathic fillets and papers with subjective analysis (WB scoring by hand palpation) because the subjective analysis is outside the aims of the present study.

### 2.2. Databases and Search Criteria

**Figure 3:**
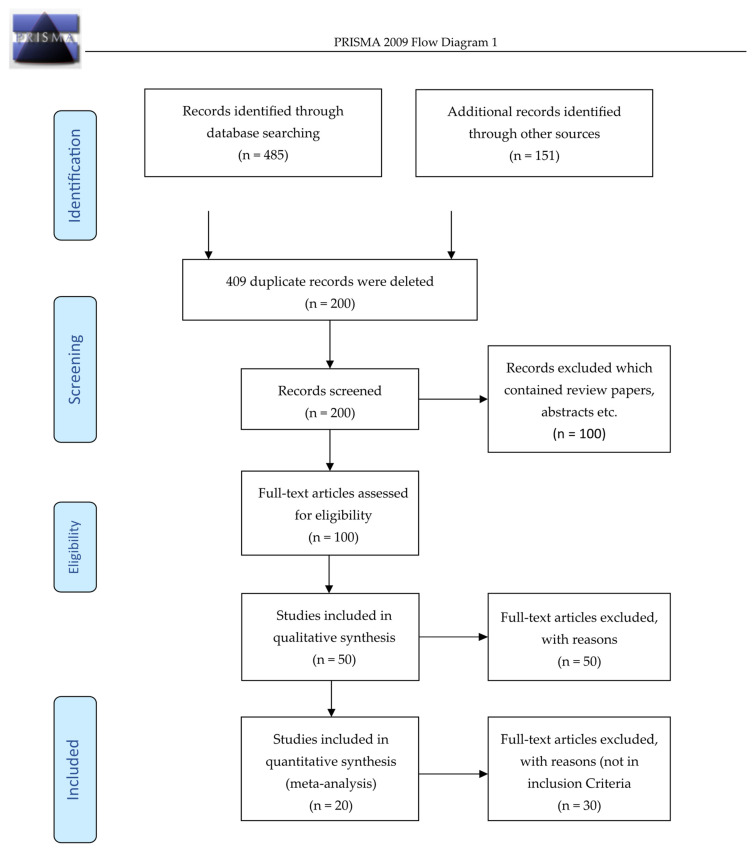
**PRISMA flow diagram for the selection of studies, inclusion and exclusion criteria**.

The Web of Science database, Google Scholar, and publications from Poultry Sciences-related journals were searched for articles that had examined the detection of woody breast condition, different cooking conditions, storage conditions, and deboning time for the normal and woody breast chicken fillets (from January 2011 to December 2021). These databases were selected on the merits of having full-text articles that were published in English. The following search string was used to locate plausible studies on woody breast myopathic conditions: “woody breast” OR “woody breast myopathies” OR “muscle abnormalities” OR “abnormalities in fast-growing broilers” OR “BMORS (Blunt Meullenet-Owens Razor Shear)” OR “MORS (Meullenet-Owens Razor Shear)” OR “TPA” (texture profile analysis) OR “compression force” OR “shear force” OR “classification accuracy” combined with different cooking and storage methods such as “Raw Frozen” OR “Frozen Thawed”, “Cooked” OR “Grilled” OR “Baked” OR “Boiled”. These searches led to 630 publications in total, from which 200 duplicate articles were removed. Other information that appeared during the search process, such as studies related to spaghetti meat, white stripping meat, and woody breast scoring by hand palpation, has been excluded to ensure a convenient search of papers related to the review questions of the paper. Full texts that were downloaded have been inspected in detail. A total of 20 complete research articles were selected on the basis of classification accuracy, toughness, tenderness, and descriptive sensory evaluation parameters such as hardness, cohesiveness, gumminess, and chewiness as analyzed using big data analytics approaches such as Support Vector Machines (SVM), Back Propagation Neural Network (BPNN), Random Forest (RF), Multilayer Perceptron (MLP), BMORS, MORS, and textural profile analysis (TPA) by descriptive sensory evaluation for age of bird, deboning time, cooking methods, and storage conditions on woody breast myopathic conditions. Every selected research article for this paper was thoroughly reviewed by other authors (B.W., and T.B.) included in this paper using standard procedure as described below (Table 1). Complete references were collected, and information that was extracted from the publication is cross-verified to determine whether the collected information was extracted from primary experimental research or from a review or meta-analysis (PRISMA flow diagram 1; Figure 3).

### 2.3. Effect Size Calculations

Effect size in meta-analysis can be defined as the difference between two experimentally created groups (control and treatment group) [46]. For this paper, the standardized mean difference (differences in means; Hedges’ g) was used to measure the difference between mean values in control (normal breast fillets) and treatment group (woody breast fillets) relative to the pooled standard deviation. This standard statistic measures how much the treatment affects the outcome on average relative to the control [46].

### 2.4. Publication Bias

The Egger’s test is used to identify funnel plot asymmetry, which is indicative of the presence of publishing bias. This test evaluates the correlation between the magnitudes of the effects and their standard errors using a linear regression methodology. A *p*-value below a certain threshold (e.g., *p* < 0.05) indicates the existence of publication bias caused by small-study effects, where smaller studies may report effect sizes that are more extreme [47]. Peters’ test is a valuable technique for identifying publication bias, especially in meta-analyses that deal with binary outcomes. This test utilizes a weighted linear regression model to analyze the correlation between the effect sizes and the sampling variances. A substantial *p*-value in Peters’ test also indicates probable bias, akin to Egger’s test but with potentially higher sensitivity for specific datasets [48]. Begg’s test is a statistical test that assesses the correlation between the effect estimates and their variances. It is a non-parametric rank correlation test. This technique is specifically designed to identify any bias that may arise from the selective publication of research that has notable findings [49]. While Egger’s test is more sensitive than Begg’s test, the latter offers an additional viewpoint on the existence of bias. The Trim and Fill method was employed to address publication bias by estimating and filling in missing studies that may have been excluded from the meta-analysis due to publication bias. This approach alters the funnel plot and recalculates the total impact size by incorporating these imputed studies [50]. The inclusion and quantity of imputed studies offer valuable information regarding the degree of bias and its influence on the outcomes of the meta-analysis. These statistical tests collectively provide a thorough evaluation of publication bias, enabling the identification and possible correction of biases that may impact the meta-analysis findings.

## 3. Data Analysis

For the analysis of collected data, R language software (Version 4.2.0; Vigorous Calisthenics) was used. Random or mixed effect models were used because the fixed effect model analyzes true effect size based on differences between studies other than one true effect size as assumed in the fixed effect model. Heterogeneity was also calculated to understand the variances in studies. A meta-regression model was used to determine the variation in effect sizes in studies that attributed to differences in classification accuracy, compression force, shear force, MORS, and BMORS due to different deboning times and ages of birds. Heterogeneity is explained by the moderator (*Q*_M_) and ominous (*Q*_E_) heterogeneity (Table 2).

## 4. Results

### 4.1. Effect of Deboning Time

In Table 3, the overall effect of deboning time had a significant impact on the different parameters evaluated for woody breast compared to the control group using the standardized mean difference (Hedges’ g [95% CI] =1.30 [0.26, 2.34], I^2^ = 95%, *p* < 0.01) and showed a strong relationship between deboning time of chicken carcasses and different parameters analyzed. The overall effect of deboning time on BMORS values is significantly different (Hedges’ g [95% CI] =0.49 [0.09, 0.89], 2.88], I^2^ = 73%, *p* < 0.01). The BMORS value for deboning time at 3 h showed a small positive effect on myopathic fillets (Hedges’ g = 0.36 [−0.23, 0.95], I^2^ = 71%, *p* < 0.01), for 2-hour deboning (Hedges’ g = 1.11 [0.30, 1.93], I^2^= *NA*), and for 8 h, a medium positive effect was observed (Hedges’ g = 0.60 [−0.39, 1.58], I^2^ = 83%, *p* < 0.01) with 83% of heterogeneity. Overall standardized mean difference for MORS analysis value (Hedges’ g = 0.70 [−0.70, 2.09], I^2^ = 95%, *p* < 0.01) showed medium effect (g ≥ 0.5) on the effect of deboning time on MORS value, high effect (g ≥ 0.8) relationship was observed for 3 h deboning time (Hedges’ g = 3.23 [−2.20, 8.66], I^2^ = 92%, *p* < 0.01) showing that MORS analysis provides better results for, negative medium effect (g ≥ 0.5) relationship was observed on 6 h deboning time (Hedges’ g = −0.71 [−1.97, 0.55], I^2^ = 83%, *p* < 0.01) and MORS value, and positive effect on 6 h deboning time (Hedges’ g = 0.36 [−0.23, 0.95], I^2^ = 71%, *p* < 0.01). For classification accuracy-based studies, analysis showed a small effect (g ≤ 0.2) (Hedges’ g [95% CI] = 0.20 [−1.35, 1.74], I^2^ = 98%, *p* < 0.01), with a positive small effect (g ≤ 0.5) for 3 h (Hedges’ g [95% CI] =0.49 [−0.67, 1.65], I^2^ = 82%, *p* < 0.01), indicating that techniques employed for the classification work performed well up to some extent for 3 h of deboning time. Overall shear force value showed negative small effect (g ≤ 0.2) for deboning time (Hedges’ g [95% CI] =−0.23 [−1.43, 0.96], I^2^ = 97%, *p* < 0.01), 3-hour deboning time favors 68.30% of studies for normal fillets analysis using shear force method (Hedges’ g [95% CI] = −0.39 [−2.24, 1.45], I^2^ = 97%, *p* < 0.01). Overall, descriptive TPA showed better meat qualities for normal fillets (g ≤ 0.2) (Hedges’ [95% CI] = −0.11 [−2.17, 1.94], I^2^ = 79%, *p* < 0.01). The majority of studies (72.30%) comparing descriptive sensory analysis for myopathic fillets with normal fillets favored 3 h of deboning time for normal fillets (Hedges’ [95% CI] = −0.41 [−3.54, 2.72], I^2^ = 84%, *p* < 0.01). Overall, textural profile analysis performed on normal and woody breast fillets for the effect of deboning time showed a significant difference in TPA values for different textural attributes (Hedges’ g [95% CI] =−0.82 [−0.14, 1.79], I^2^ = 83%, *p* <0.01). TPA values when analyzed separately for 2 h (Hedges’ g [95% CI] =−0.04 [−0.13, 0.21], I^2^ = 29%, *p* = 0.19) were not significantly different when compared to 3 h (Hedges’ g [95% CI] =1.11 [−0.20, 2.42], I^2^ = 85%, *p* < 0.01) deboning time.

### 4.2. Effect on the Age of Birds

In the given Table 4 below, the breast fillets that are analyzed for MORS (pooled Hedges’ g [95% CI] = 0.70 [−0.70, 2.09], I^2^ = 95%, *p* < 0.01), BMORS (pooled Hedges’ g [95% CI] = 0.49 [0.09, 0.89], I^2^ = 73%, *p*< 0.01), shear force (pooled Hedges’ g [95% CI] = −0.23 [−1.43, 0.96], I^2^ = 97%, *p* < 0.01), classification accuracy (pooled Hedges’ g [95% CI] = 0.20 [−1.35, 1.74], I^2^ = 98%, *p* < 0.01) and for the descriptive analysis (TPA) (pooled Hedges’g [95% CI] = −0.09 [−2.13, 1.94], I^2^ = 79%, *p* < 0.01) were significantly different for processing age of birds. From the analysis, large effects (g > 0.8) were observed for compression force for all ages of birds ranging from 34 to 56 days old. Small effects (g < 0.5) were observed for 56-day-old birds when classification accuracy for rapid detection approaches, conventional BMORS analysis, and MORS analysis were performed on the effect of age of birds. Overall, negative Hedges’ g values for shear force and descriptive analysis showed a small effect (g < 0.2), based on the individual age of birds. Moreover, 45-day-old birds showed large effects (g > 0.8) for shear force and descriptive sensory analysis. The overall effect of age shows a significant effect on the breast meat quality (Hedges’ g [95% CI] = 1.30 [0.26, 2.34], I^2^ = 95%, *p* < 0.01). When analyzed together for classification accuracy, compression force, shear force, BMORS, MORS, and TPA (descriptive analysis), the Hedges g values for age of birds: at 34 days old (Hedges’ g [95% CI] = 1.43 [−2.06, 4.92], I^2^ = 91%, *p* < 0.01), at 38 days old (Hedges’ g [95% CI] = 11.05 [−108.57, 132.28], I^2^ = 95%, *p* = 0.58), at 42 days old (Hedges’ g [95% CI] = 0.39 [0.09, 0.68], I^2^ = 0.00%, *p* = 0.86), at 45 days old (Hedges’ g [95% CI] = 2.29 [−0.49, 5.06], I^2^ = 86%, *p* < 0.01), 46 days old (Hedges’ g [95% CI] = 1.05 [0.73, 1.37], I^2^ = *NA*), 48 days old (Hedges’ g [95% CI] = 0.27 [−1.02, 1.57], I^2^ = 96%, *p* < 0.01), 52 days old (Hedges’ g [95% CI] = 0.63 [−15.62, 16.87], I^2^ = 98%, *p* < 0.01), 56 days old (Hedges’ g [95% CI] = 1.10 [−0.63, 2.83], I^2^ = 96%, *p* < 0.01), and at 60 days old (Hedges’ g [95% CI] = 0.03 [−0.10, 0.17], I^2^ = 0.00%, *p* = 0.58) respectively, indicating that birds at the ages of 34 d, 38 d, 45 d, 46 d, and 56 d showed a large effect (g > 0.8), birds at the age of 52 d showed medium (g > 0.5), while birds at the age of 42 d, 48 d, and 60 days showed small effect on different parameters evaluated on bird’s age. Birds that are processed at the age of 45 days (Hedges’ g [95% CI] = 2.66 [−0.86, 6.18], I^2^ = 92%, *p* < 0.01) and 52 days (Hedges’ g [95% CI] = 0.53 [−0.27, 1.33], I^2^ = 74%, *p* < 0.01) are significantly different from other processed birds at the age of 42, 56, and 60 days, respectively.

### 4.3. Effect of Different Storage and Cooking Conditions

In Table 5, the different storage conditions of raw and further processed fillets and In Table 5, the different storage conditions of raw and further processed fillets and their products are key factors in affecting the quality parameters such as texture (toughness, tenderness, juiciness, and chewiness), appearance (color), odor, and overall acceptability of the product. It also affects the chemical parameters related to the meat quality [26,28]. The overall effect of different cooking conditions (Hedges’ g [95% CI] = 0.72 [0.17, 1.26], I^2^ = 93%, *p* < 0.01) has significant effects on breast fillet quality. Cooked breast fillets (Hedges’ g [95% CI] = 0.44 [0.21, 0.67], I^2^ = 54%, *p* < 0.01) showed a significant effect on shear force energies values obtained from MORS, BMORS, and sensory descriptive evaluation values. There were no significant differences observed between cooked hot served (Hedges’ g [95% CI] =− 0.09 [−0.44, 0.26], I^2^ = 41%, *p* < 0.17) and cooked cold served (Hedges’ g [95% CI] = 0.17 [0.13, 0.21], I^2^ = 0%, *p* < 0.99) breast fillets to the sensory panel for descriptive sensory evaluation. BMORS shear force values for cooked breast fillets were not significant when compared to raw breast fillets (Hedges’ g [95% CI] = 0.69 [−0.22, 1.60], I^2^ = 98%, *p* < 0.01). Overall, BMORS shear force values for the cooking conditions were significantly different (Hedges’ g [95% CI] = 1.07 [−0.73, 2.88], I^2^ = 97%, *p* < 0.01). MORS shear force value cooked samples (Hedges’ g [95% CI] = 0.93 [−0.10, 7.87], I^2^ = 85%, *p* = 0.01) were significantly different for various cooking methods in work conducted by Combs [38], such as baked, cooked frozen, sous vide, grill, and raw frozen. Overall, the sous vide method of cooking for woody breast fillets showed a significant effect for different analyzed shear force energies and sensory descriptive sensory evaluation (Hedges’ g [95% CI] = 5.30 [−50.30, 83.40], I^2^ = 98%, *p* < 0.01).

### 4.4. Publication Bias

Based on the analysis presented in Table 6, publication bias can be observed across various analyzed attributes. The TPA_Hardness results showed bias in all the conducted tests (Egger’s test *p*-value of 0.0182, Peters’ test *p*-value of 0.0003, and Begg’s test *p*-value of 0.0446). The Trim and Fill method did not find any missing studies, indicating that the observed bias is likely inherent rather than a result of missing data. This has important implications for the study’s findings, highlighting the significance of bias analysis. For TPA_Springiness, less publication bias was observed. Egger’s test (*p* = 0.1932) and Peters’ test (*p* = 0.0978) both suggested a low bias. On the other hand, Begg’s test indicated a slight bias with a *p*-value of 0.0752. One missing study was identified by the Trim and Fill method, indicating that there may be some bias in this attribute. The TPA_Cohesiveness data exhibited a clear publication bias, as indicated by the results of Peters’ test (*p* < 0.0001). However, the findings from Egger’s test (*p* = 0.1228) and Begg’s test (*p* = 0.3585) did not yield statistically significant results. This difference in results suggests that Peters’ test may be more effective in identifying bias related to this attribute. TPA_Chewiness showed a clear bias, as confirmed by Egger’s test (*p* = 0.0082) and Peters’ test (*p* < 0.0001). No additional studies were included, indicating that bias is inherent in the data. MORS_Tenderness displayed a slight or borderline bias, with only Begg’s test (*p* = 0.0117) showing significance. There were noticeable biases found in all of the tests conducted for BMORS_Toughness. The results from Egger’s test (*p* < 0.0001) and Peters’ test (*p* = 0.0090) were particularly strong. Additionally, the Trim and Fill method identified one study that was missing. Cookloss_ also showed a clear bias, as demonstrated by the results of Egger’s (*p* = 0.0328) and Peters’ tests (*p* < 0.0001), and no other studies were found.

## 5. Discussion

The primary objective of this study was to assess the influence of deboning time, bird age, and various cooking conditions on the precision of categorization, compression force, shear force, and sensory evaluations in both normal and woody breast fillets. The findings revealed that diverse cooking settings have a substantial impact on shear force values and sensory descriptive analysis [51]. However, the age of the bird and the duration of deboning did not notably affect the shear force values of BMORS when comparing woody breasts to normal fillets. The impact of cooking settings on BMORS shear force was significant, as indicated by *p*-values ranging from 0.01 to 0.001. In contrast, the MORS shear force values remained unaffected by bird age and cooking circumstances. Nevertheless, the duration of deboning had a significant influence.

Minimal impacts of deboning time, chicken age, and cooking parameters were noted on sensory assessments when comparing woody breast fillets. This research validates previous findings by demonstrating that heating substantially impacts the texture of meat. This is primarily caused by the denaturation of proteins and fats, resulting in muscle toughening and higher shear values [51]. The analysis showed that the cooking conditions significantly impacted the ability of shear force measurements to differentiate between different woody breast groups [52].

In summary, the analysis revealed the presence of variability in shear force values, which may be attributed to factors such as marination time, procedures used, and the physiochemical properties of the fillets. The accuracy of classification was affected by the duration of deboning and the analytical procedures employed, with variations associated with factors such as collagen composition and data processing methodologies [53]. The work underlines the intricate nature of classification problems, in which factors such as data linearity, preprocessing, and unknown confounders have substantial influences. In addition, fast-growing broiler birds are more susceptible to woody breast, and deboning the meat early resulted in higher shear force values. The study addresses essential questions regarding the most effective cooking settings and deboning durations that achieve a balance between processing efficiency and product quality [5,54].

## 6. Conclusions

In conclusion, this meta-analysis provides evidence that there are very small numbers of published studies available for a comparative study between normal and woody breast fillets due to the fact that there is not a fixed quantitative method for the classification of myopathic fillets, and the methods that are available for the classification are completely based on employee experiences, which are more susceptible to giving deviated false results during the processes. Other factors that contribute to these results are unexperienced employees, speed of processing lines, stress on employees, and levels of fatigue. In our observation, those studies that have used big data analytic approaches such as regression models, LDAs, and computer vision systems have mainly focused on the identification techniques and reported whether the implemented techniques are able to detect the myopathic conditions in fillets or not without performing comparisons by how much the new technique is able to detect these conditions. More studies are encouraged to be performed to explore different methods to classify the fillets based on quantitative methods rather than qualitative approaches to set a standardized parameter with new innovative technologies in poultry processing plants that can be placed to reduce the losses associated with the misclassification of these fillets and will also be helpful in maintaining the quality and keeping up with the speed that can reduce the incidences of misclassification. Interestingly, studies that have used myopathic fillets in further processing products that utilize the woody breast fillets have agreed to the fact that further processing steps for different products made from these myopathic fillets do not differ from the normal fillet product, and the consumer panel found these products as acceptable in nature.

## Figures and Tables

**Table 1:** Summary table for the studies used in the analysis.

Reference No.	Study	Deboning Time (h)	Meat Condition and Cooking Style	Analysis Method	Age of Birds (Days)
[28]	Lee et al., 2014	0.25, 1.25, 2.0, 2.5, 3.0, 3.5, 4.0, 6.0, and 24.0	Water Cooking and Oven-Baking	MORS and Descriptive analysis	42
[29]	Yang, 2016	2, 4, 6, and 24	Fillets and Conventional Oven	MORS and BMORS	40 and 54
[30]	Tijare et al., 2016	2 and 4	Air Convection Oven	MORS	42 and 63
[31]	Chatterjee et al., 2016	3	Raw and Cooked Fillets	MORS and Textural Profile Analysis	56
[32]	Solo, 2016	2	Air Convection Oven	MORS, Compression Force, and BMORS	45, 63, and 70
[33]	Cando, 2016	3	Flat top grill or in an Air Convection Oven	Textural Profile Analysis and Shear Force	52
[22]	Soglia et al., 2016	3	Air Convection Oven	Texture Profile Analysis	52
[34]	U-Chupaj et al., 2017	6	Cooked fillets	Shear Force and Textural Profile Analysis	56
[35]	Brambila et al., 2017	3	Fillets and Patties	Shear force and Textural Profile Analysis	56
[36]	Aguirre et al., 2018	3	Air Convection Oven	Textural Profile Analysis	45
[37]	Brambila et al., 2018	3	Air Convection Oven	Texture Profile Analysis	42
[38]	Combs, 2018	6	Raw, Grill, Bake, and Sous vide	MORS and BMORS	56
[39]	Bowker and Zhuang, 2019	8	Raw Frozen, Cooked Frozen, Raw, and Cooked	MORS and BMORS	56
[40]	Pang et al., 2020	3	Raw Fillets and Air Convection Oven	MORS and BMORS	56
[41]	Mallmann et al., 2020	3	Raw Fillets and Conventional oven	Compression force, MORS, and BMORS	57
[42]	Morey et al., 2020	2–3	Fillets and Conventional Oven	BMORS	56
[43]	Zhang et al., 2021	3	Raw and Conventional Oven	Shear Force, Compression Force, and BMORS	56
[44]	Sun et al., 2021	3	Fillets and Conventional Oven	BMORS	49
[45]	Sun et al., 2021	3	Raw Fillets and Conventional Oven	Compression Force and BMORS	49
[18]	Siddique et al., 2021	3–3.5	Raw Fillets	Bioelectrical Impedance, Support Vector Machines, and Back Propagation Neural Networks	56

**Table 2:** Meta-regression of moderators including age of bird, deboning time, and condition (cooking and storage) on the shear force of MORS, BMORS, and other textural parameters analyzed by descriptive sensory evaluation.

Analysis Type	Parameters	*Q*_E_	df	*p*	*Q*_M_	df	*p*	τ^2^	I^2^
	Age of Bird	335.02	8	<0.01	0.54	5	0.99	14.55	99.65
BMORS	Deboning Time	274.59	9	<0.01	1.02	4	0.90	12.17	99.69
	Cooking and Storage	330.30	7	<0.01	60.60	7	<0.001	1.16	98.01
	Age of Bird	181.02	15	<0.01	3.37	5	0.64	0.52	91.34
MORS	Deboning Time	64.85	15	<0.01	32.13	5	<0.0001	0.17	77.97
	Cooking and Storage	273.78	14	<0.01	4.84	6	0.56	0.51	93.44
	Age of Bird	176.44	31	<0.01	14.33	4	0.0063	0.33	83.75
Descriptive Sensory	Deboning Time	202.39	34	<0.01	3.10	1	0.07	0.44	86.93
	Cooking and Storage	129.79	27	<0.01	51.83	8	<0.0001	0.33	83.81

***Q*_E_ (Q for error)** represents the residual heterogeneity in the effect sizes after accounting for moderators. **df (degrees of freedom):** Refers to the degrees of freedom associated with the *Q*_E_ and *Q*_M_ tests. In the context of *Q*_E_, it typically equals the number of studies minus the number of parameters estimated. ***p* (*p*-value):** Indicates the significance level of the test statistics. A *p*-value less than 0.05 suggests statistically significant results. ***Q*_M_ (Q for Model):** Tests whether the moderators significantly explain the variance in effect sizes across studies. A significant *Q*_M_ value implies that the moderators included in the model have a substantial impact. **τ^2^ (Tau-squared):** Estimates the between-study variance in a random-effects model, reflecting the extent of variability among the true effect sizes beyond sampling error. **I^2^ (I-squared):** Measures the proportion of total variation in effect sizes due to heterogeneity rather than chance, expressed as a percentage. Higher I^2^ values indicate greater heterogeneity.

**Table 3:** Summary table for the analysis based on effect of deboning time.

Parameter	Deboning Time	Effect Size (Hedges’ g [95% CI])	I^2^ (%)	*p*-Value
Overall Effect of Deboning Time	Overall	1.30 [0.26, 2.34]	95	<0.01
BMORS Values	Overall	0.49 [0.09, 0.89]	73	<0.01
	3 h	0.36 [−0.23, 0.95]	71	<0.01
	2 h	1.11 [0.30, 1.93]	NA	<0.01
	8 h	0.60 [−0.39, 1.58]	83	<0.01
MORS Analysis Values	Overall	0.70 [−0.70, 2.09]	95	<0.01
	3 h	3.23 [−2.20, 8.66]	92	<0.01
	6 h	−0.71 [−1.97, 0.55]	83	<0.01
	6 h	0.36 [−0.23, 0.95]	71	<0.01
Classification Accuracy	Overall	0.20 [−1.35, 1.74]	98	<0.01
	3 h	0.49 [−0.67, 1.65]	82	<0.01
Shear Force Value	Overall	−0.23 [−1.43, 0.96]	97	<0.01
	3 h	−0.39 [−2.24, 1.45]	97	<0.01
Descriptive TPA	Overall	−0.11 [−2.17, 1.94]	79	<0.01
Descriptive Sensory Analysis	3 h	−0.41 [−3.54, 2.72]	84	<0.01
Textural Profile Analysis (TPA)	Overall	−0.82 [−0.14, 1.79]	83	<0.01
	2 h	−0.04 [−0.13, 0.21]	29	0.19
	3 h	1.11 [−0.20, 2.42]	85	<0.01

**Hedges’ g:** Hedges’ g is an effect size metric that measures the difference between two group means, adjusted for sample size bias. It is used to estimate the magnitude of the effect or difference between a control group and a treatment group, with values typically interpreted as follows: 0.2 = small effect, 0.5 = medium effect, and 0.8 = large effect. **The confidence interval (CI)**: The CI value provides a range within which the true effect size is likely to fall. **I^2^ (%):** I^2^ is a statistical measure used to quantify the degree of heterogeneity in meta-analyses. It represents the percentage of variation across studies that is due to heterogeneity rather than chance. Values of 25%, 50%, and 75% are considered to indicate low, moderate, and high heterogeneity, respectively. A higher I^2^ suggests greater variability among study outcomes, which may reflect differences in study design, populations, or other factors.

**Table 4:** Summary table for the analysis on effect of age of bird on different parameters.

Bird Age (Days)	Parameter	Hedges’ g [95% CI]	I^2^ (%)	*p*-Value
34	Classification Accuracy, Compression Force, Shear Force, BMORS, MORS, and TPA	1.43 [−2.06, 4.92]	91	<0.01
38	Classification Accuracy, Compression Force, Shear Force, BMORS, MORS, and TPA	11.05 [−108.57, 132.28]	95	0.58
42	Classification Accuracy, Compression Force, Shear Force, BMORS, MORS, and TPA	0.39 [0.09, 0.68]	0.00	0.86
45	Classification Accuracy, Compression Force, Shear Force, BMORS, MORS, and TPA	2.29 [−0.49, 5.06]	86	<0.01
46	Classification Accuracy, Compression Force, Shear Force, BMORS, MORS, and TPA	1.05 [0.73, 1.37]	NA	NA
48	Classification Accuracy, Compression Force, Shear Force, BMORS, MORS, and TPA	0.27 [−1.02, 1.57]	96	<0.01
52	Classification Accuracy, Compression Force, Shear Force, BMORS, MORS, and TPA	0.63 [−15.62, 16.87]	98	<0.01
56	Classification Accuracy, Compression Force, Shear Force, BMORS, MORS, and TPA	1.10 [−0.63, 2.83]	96	<0.01
60	Classification Accuracy, Compression Force, Shear Force, BMORS, MORS, and TPA	0.03 [−0.10, 0.17]	0.00	0.58

**Hedges’ g:** Hedges’ g is an effect size metric that measures the difference between two group means, adjusted for sample size bias. It is used to estimate the magnitude of the effect or difference between a control group and a treatment group, with values typically interpreted as follows: 0.2 = small effect, 0.5 = medium effect, and 0.8 = large effect. **The confidence interval (CI)**: The CI value provides a range within which the true effect size is likely to fall. **I^2^ (%):** I^2^ is a statistical measure used to quantify the degree of heterogeneity. It represents the percentage of variation across studies that is due to heterogeneity rather than chance. Values of 25%, 50%, and 75% are considered to indicate low, moderate, and high heterogeneity, respectively. A higher I^2^ suggests greater variability among study outcomes, which may reflect differences in study design, populations, or other factors.

**Table 5:** Summary table for the analysis conducted for effect of cooking conditions on different parameters.

Cooking Condition	Parameter	Hedges’ g [95% CI]	I^2^ (%)	*p*-Value
Overall Effect	All Parameters	0.72 [0.17, 1.26]	93	<0.01
Cooked Breast Fillets	Shear Force	0.44 [0.21, 0.67]	54	<0.01
Cooked Hot Served	Descriptive Sensory Evaluation	−0.09 [−0.44, 0.26]	41	0.17
Cooked Cold Served	Descriptive Sensory Evaluation	0.17 [0.13, 0.21]	0	0.99
BMORS Shear Force (Cooked vs. Raw)	Shear Force	0.69 [−0.22, 1.60]	98	<0.01
BMORS Shear Force (Overall Cooking Conditions)	Shear Force	1.07 [−0.73, 2.88]	97	<0.01
MORS Shear Force (Cooked Samples)	Shear Force	0.93 [−0.10, 7.87]	85	0.01
Sous Vide Method	Shear Force and Descriptive Sensory Evaluation	5.30 [−50.30, 83.40]	98	<0.01

**Hedges’ g:** Hedges’ g is an effect size metric that measures the difference between two group means, adjusted for sample size bias. It is used to estimate the magnitude of the effect or difference between a control group and a treatment group, with values typically interpreted as follows: 0.2 = small effect, 0.5 = medium effect, and 0.8 = large effect. **The confidence interval (CI)**: The CI value provides a range within which the true effect size is likely to fall. **I^2^ (%):** I^2^ is a statistical measure used to quantify the degree of heterogeneity. It represents the percentage of variation across studies that is due to heterogeneity rather than chance. Values of 25%, 50%, and 75% are considered to indicate low, moderate, and high heterogeneity, respectively. A higher I^2^ suggests greater variability among study outcomes, which may reflect differences in study design, populations, or other factors.

**Table 6:** Summary table for the publication bias analysis using Egger’s test, Peter’s test, Begg’s test, and the Trim and Fill approach.

Method and Attribute	Egger’s Test (*p*-Value)	Peters’ Test (*p*-Value)	Begg’s Test (*p*-Value)	Trim and Fill (Imputed Studies)	Presence of Bias
TPA_Hardness	0.0182	0.0003	0.0446	0	Yes
TPA_Springiness	0.1932	0.0978	0.0752	1	Mild/Borderline
TPA_Cohesiveness	0.1228	<0.0001	0.3585	0	Yes
TPA_Chewiness	0.0082	<0.0001	0.1802	0	Yes
MORS_Tenderness	0.5925	0.1205	0.0117	0	Mild/Borderline
BMORS_Toughness	<0.0001	0.0090	0.0833	1	Yes
Cookloss_	0.0328	<0.0001	0.3333	0	Yes

## Data Availability

No new data were created or analyzed in this study. Data sharing is not applicable to this article.

## References

[1] Heinz G., Hautzinger P. (2020). Meat Processing Technology. For Small to Medium-Scale Producers. Food and Agriculture Organization United Nations Regional Office Asia Pacific. https://openknowledge.fao.org/server/api/core/bitstreams/4cfabbd3-16aa-47f8-ac6f-b54a48cb8abd/content.

[2] National Chicken Council Statistics: Broiler Chicken Industry Key Facts 2022. http://www.nationalchickencouncil.org/about-the-industry/statistics/broiler-chicken-industry-key-facts/.

[3] Maiorano G. (2017). Meat defects and emergent muscle myopathies in broiler chickens: Implications for the modern poultry industry. Anim. Sci. Genet..

[4] Petracci M., Bianchi M., Mudalal S., Cavani C. (2013). Functional ingredients for poultry meat products. Trends Food Sci. Technol..

[5] Petracci M., Cavani C. (2012). Muscle growth and poultry meat quality issues. Nutrients.

[6] Sihvo H., Immonen K., Puolanne E. (2014). Myodegeneration with fibrosis and regeneration in the pectoralis major muscle of broilers. Vet. Pathol..

[7] Che S., Wang C., Varga C., Barbut S., Susta L. (2022). Prevalence of breast muscle myopathies (spaghetti meat, woody breast, white striping) and associated risk factors in broiler chickens from Ontario, Canada. PLoS ONE.

[8] Kuttappan V.A., Owens C.M., Coon C., Hargis B.M., Vazquez-Anon M. (2017). Incidence of broiler breast myopathies at 2 different ages and its impact on selected raw meat quality parameters. Poult. Sci..

[9] Velleman S.G., Clark D.L., Tonniges J.R. (2017). Fibrillar collagen organization associated with broiler wooden breast fibrotic myopathy. Avian Dis..

[10] Soglia F., Gao J., Mazzoni M., Puolanne E., Cavani C., Petracci M., Ertbjerg P. (2017). Superficial and deep changes of histology, texture, and particle size distribution in broiler wooden breast muscle during refrigerated storage. Poult. Sci..

[11] Kuttappan V., Lee Y., Erf G., Meullenet J., McKee S., Owens C. (2012). Consumer acceptance of visual appearance of broiler breast meat with varying degrees of white striping. Poult. Sci..

[12] Chicco D., Jurman G. (2020). The advantages of the Matthews correlation coefficient (MCC) over F1 score and accuracy in binary classification evaluation. BMC Genom..

[13] Huang Y., Kangas L.J., Rasco B.A. (2007). Applications of artificial neural networks (ANNs) in food science. Crit. Rev. Food Sci. Nutr..

[14] Cortez P., Portelinha M., Rodrigues S., Cadavez V., Teixeira A. (2006). Lamb meat quality assessment by support vector machines. Neural Process. Lett..

[15] Asmara R.A., Rahutomo F., Hasanah Q., Rahmad C. (2017). Chicken meat freshness identification using the histogram color feature. Proceedings of the 2017 International Conference on Sustainable Information Engineering and Technology (SIET).

[16] Ning X., Mu-Hua L., Hai-Chao Y., Shuang-Gen H., Xiao W., Jin-Hui Z., Yi-Xin S. (2020). Classification of sulfadimidine and sulfapyridine in duck meat by surface-enhanced Raman spectroscopy combined with principal component analysis and support vector machine. Anal. Lett..

[17] Rumelhart D.E., McClelland J.L. (1988). PDP Research Group. Parallel Distributed Processing.

[18] Siddique A., Shirzaei S., Smith A.E., Valenta J., Garner L.J., Morey A. (2021). Acceptability of Artificial Intelligence in Poultry Processing and Classification Efficiencies of Different Classification Models in the Categorisation of Breast Fillet Myopathies. Front. Physiol..

[19] Dalgaard L.B., Rasmussen M.K., Bertram H.C., Jensen J.A., Møller H.S., Aaslyng M.D., Young J.F. (2018). Classification of wooden breast myopathy in chicken pectoralis major by a standardized method and association with conventional quality assessments. Int. J. Food Sci. Technol..

[20] Sun X., Koltes D.A., Coon C.N., Chen K., Owens C.M. (2018). Instrumental compression force and meat attribute changes in woody broiler breast fillets during short-term storage. Poult. Sci..

[21] Mudalal S., Lorenzi M., Soglia F., Cavani C., Petracci M. (2015). Implications of white striping and wooden breast abnormalities on quality traits of raw and marinated chicken meat. Animal.

[22] Soglia F., Mudalal S., Babini E., Di Nunzio M., Mazzoni M., Sirri F., Petracci M. (2016). Histology, composition, and quality traits of chicken pectoralis major muscle affected by wooden breast abnormality. Poult. Sci..

[23] Destefanis G., Brugiapaglia A., Barge M.T., Dal Molin E. (2008). Relationship between beef consumer tenderness perception and Warner–Bratzler shear force. Meat Sci..

[24] Dar Y.L., Light J.M. (2014). Food Texture Design and Optimization.

[25] Cavitt L.C., Meullenet J.F., Gandhapuneni R.K., Youm G.W., Owens C.M. (2005). Rigor development and meat quality of large and small broilers and the use of Allo-Kramer shear needle puncture and razor blade shear to measure texture. Poult. Sci..

[26] Meullenet J.F., Jonville E., Grezes D., Owens C.M. (2004). Prediction of the texture of cooked poultry pectoralis major muscles by near-infrared reflectance analysis of raw meat. J. Texture Stud..

[27] ASTM (1992). Manual on Descriptive Analysis Testing for Sensory Evaluation.

[28] Lee Y.S., Owens C.M., Meullenet J.F. (2008). The Meullenet-Owens razor shear (MORS) for predicting poultry meat tenderness: Its applications and optimization. J. Texture Stud..

[29] Yang F.L. (2016). Use of a Blunt Version of Meullenet-Owens Razor Shear to Analyze Meat Qualities of Broilers with Woody Breast Myopathy and Reared to Various Ages. Master’s Thesis.

[30] Tijare V.V., Yang F.L., Kuttappan V.A., Alvarado C.Z., Coon C.N., Owens C.M. (2016). Meat quality of broiler breast fillets with white striping and woody breast muscle myopathies. Poult. Sci..

[31] Chatterjee D., Zhuang H., Bowker B.C., Sanchez-Brambila G., Rincon A.M. (2016). Instrumental texture characteristics of broiler pectoralis major with the wooden breast condition. Poult. Sci..

[32] Solo J. (2016). Meat Quality and Sensory Analysis of Broiler Breast Fillets with Woody Breast Muscle Myopathy. Master’s Thesis.

[33] Aguirre Cando M.E. (2016). Study of the Woody Breast and White Striping Conditions Affecting the Pectoralis Major Muscle of Broiler Chickens: Processing Perspective. Ph.D. Thesis.

[34] U-Chupaj J., Malila Y., Gamonpilas C., Kijroongrojana K., Petracci M., Benjakul S., Visessanguan W. (2017). Differences in textural properties of cooked caponized and broiler chicken breast meat. Poult. Sci..

[35] Brambila G.S., Chatterjee D., Bowker B., Zhuang H. (2017). Descriptive texture analyses of cooked patties made of chicken breast with the woody breast condition. Poult. Sci..

[36] Aguirre M.E., Owens C.M., Miller R.K., Alvarado C.Z. (2018). Descriptive sensory and instrumental texture profile analysis of woody breast in marinated chicken. Poult. Sci..

[37] Brambila G.S., Bowker B.C., Chatterjee D., Zhuang H. (2018). Descriptive texture analyses of broiler breast fillets with the wooden breast condition stored at 4 °C and −20 °C. Poult. Sci..

[38] Combs L. (2018). Effect of Cooking Method on Meat Texture in Normal and Woody Broiler Breast Fillets Using Instrumental Analysis and Descriptive Sensory Analysis. Master’s Thesis.

[39] Bowker B., Zhuang H. (2019). Detection of razor shear force differences in broiler breast meat due to the woody breast condition depends on measurement technique and meat state. Poult. Sci..

[40] Pang B., Bowker B., Yang Y., Zhang J., Zhuang H. (2020). Relationships between instrumental texture measurements and subjective woody breast condition scores in raw broiler breast fillets. Poult. Sci..

[41] de Almeida Mallmann B., Tellez-Isaias G., Mauromoustakos A., Coon C.N., Owens C.M. (2020). Fillet dimensions and meat quality attributes associated with woody breast in broilers. Meat Muscle Biol..

[42] Morey A., Smith A.E., Garner L.J., Cox M.K. (2020). Application of bioelectrical impedance analysis to detect broiler breast filets affected with woody breast myopathy. Front. Physiol..

[43] Zhang J., Zhuang H., Bowker B., Stelzleni A.M., Yang Y., Pang B., Gao Y., Thippareddi H. (2021). Evaluation of multi blade shear (MBS) for determining texture of raw and cooked broiler breast fillets with the woody breast myopathy. Poult. Sci..

[44] Sun X., Giampietro-Ganeco A., Mueller A., Maynard C.J., Caldas-Cueva J.P., Owens C.M. (2021). Meat quality traits and Blunt Meullenet-Owens Razor Shear characteristics of broiler breast fillets affected by woody breast condition and post-cooking meat temperature. Poult. Sci..

[45] Sun X., Maynard C.J., Caldas-Cueva J.P., Coon C.N., Owens C.M. (2021). Using air deformation of raw fillet surfaces to identify severity of woody breast myopathy in broiler fillets. LWT.

[46] Kemp S.E., Ng M., Hollowood T., Hort J. (2018). Introduction to descriptive analysis. Descriptive Analysis in Sensory Evaluation.

[47] Morris S.B., DeShon R.P. (2002). Combining effect size estimates in meta-analysis with repeated measures and independent-groups designs. Psychol. Methods.

[48] Egger M., Smith G.D., Schneider M., Minder C. (1997). Bias in meta-analysis detected by a simple graphical test. BMJ.

[49] Peters J.L., Sutton A.J., Jones D.R., Abrams K.R., Rushton L. (2006). Comparison of two methods to detect publication bias in meta-analysis. JAMA.

[50] Begg C.B., Mazumdar M. (1994). Operating characteristics of a rank correlation test for publication bias. Biometrics.

[51] Souza P.A., Kodawara L.M., Pelicano E.R., Souza H.B., Oba A., Leonel F.R., Norkus E.A., Lima T.M. (2005). Effect of deboning time on the quality of broiler breast meat (Pectoralis major). Braz. J. Poult. Sci..

[52] Forrest J.C., Aberle E.D., Hedrick H.B., Judge M.D., Merkel R.A. (1975). Principles of meat processing. Principles of Meat Science.

[53] Duval S., Tweedie R. (2000). Trim and fill: A simple funnel-plot–based method of testing and adjusting for publication bias in meta-analysis. Biometrics.

[54] Møller A.J. (1981). Analysis of Warner-Bratzler shear pattern with regard to myofibrillar and connective tissue components of tenderness. Meat Sci..

